# The Effects of a High Concentration of Dissolved Oxygen on Actinobacteria from Lake Baikal

**DOI:** 10.3390/metabo13070830

**Published:** 2023-07-07

**Authors:** Maria E. Dmitrieva, Ekaterina V. Malygina, Alexander Y. Belyshenko, Victoria N. Shelkovnikova, Natalia A. Imidoeva, Maria M. Morgunova, Tamara Y. Telnova, Anfisa A. Vlasova, Denis V. Axenov-Gribanov

**Affiliations:** Laboratory of Experimental Neurophysiology, Department for Research and Development, Irkutsk State University, 1 Karl Marx Str., 664003 Irkutsk, Russia; marriee.dmitrieva@gmail.com (M.E.D.); cat.malygina@gmail.com (E.V.M.); al.belyshenko@gmail.com (A.Y.B.); shelkovnikova551@gmail.com (V.N.S.); nat.imidoeva@gmail.com (N.A.I.); marymikhmorg@gmail.com (M.M.M.); telnovatamara1410@gmail.com (T.Y.T.); vlasovafippo23@gmail.com (A.A.V.)

**Keywords:** actinobacteria, oxyphilic bacteria, Lake Baikal, oxygen, ROS, oxidative stress, antioxidants, secondary metabolites, DNA mutations

## Abstract

Among the diversity of microorganisms, the rarest and least explored are microorganisms that live in conditions of high oxygen in the environment and can experience the effects of natural oxidative stress. Here we suggest that the actinobacteria of Lake Baikal, sampled in the littoral zone, may produce natural products with antioxidant activity. The current study aimed to assess the effects of experimentally increased amounts of oxygen and ozone on the morphology of actinobacteria, DNA mutations, and antioxidant potential. In this experiment, we cultivated actinobacteria in liquid culture under conditions of natural aeration and increased concentrations of dissolved oxygen and ozone. Over a period of three months, bacterial samples were collected every week for further analysis. Morphological changes were assessed using the Gram method. A search for DNA mutations was conducted for the highly conserved 16S rRNA gene. The evaluation of antioxidant activity was performed using the DPPH test. The biotechnological potential was evaluated using high-resolution liquid chromatography-mass spectrometry approaches supplemented with the dereplication of natural products. We demonstrated the synthesis of at least five natural products by the *Streptomyces* sp. strain only under conditions of increased oxygen and ozone levels. Additionally, we showed morphological changes in *Streptomyces* sp. and nucleotide mutations in *Rhodococcus* sp. exposed to increased concentrations of dissolved oxygen and oxidative stress. Consequently, we demonstrated that an increased concentration of oxygen can influence Lake Baikal actinobacteria.

## 1. Introduction

Oxygen is a key environmental factor that affects the growth and physiology of organisms. For most microorganisms, it plays an essential role in metabolism, energy production, and respiration [1,2]. Microorganisms have adapted over time to live in various ecological niches that differ in oxygen levels. This adaptation has led to improvements in the respiratory chain, where oxygen serves as the final electron acceptor [3]. However, it also increases the likelihood of damage to biomolecules by its active forms [4]. Normally, the reduction of oxygen leads to the formation of water [5]. However, in some cases, the loss of electrons from intermediate carriers in the respiratory chain can result in the formation of toxic forms of oxygen and the induction of oxidative stress [6]. Oxidative stress is an excess of reactive oxygen species (ROS) compared to antioxidants and is formed as a product of normal oxygen metabolism. ROS are necessary for biological processes, while oxidative stress results from the activation of the physiological pathway of cell death [7]. An increase in the level of ROS can lead to chain reactions that damage carbohydrates, lipids, proteins, and nucleic acids [8].

It has been shown that ROS can have a mutagenic effect on various organisms, from bacteria to humans, and cause genomic instability [9]. The most harmful effects occur when ROS act on DNA molecules, leading to the damage of both nucleotides and deoxyribose residues. ROS can result in the formation of thymine glycols at the nucleotide level, leading to mutations such as the replacement of thymine with cytosine in a single DNA strand [10]. Additionally, ROS can induce the formation of 8-oxoguanine by oxidizing nucleotide bases, which can lead to transversions of guanine to cytosine or guanine to adenine [11,12]. Furthermore, high concentrations of ozone can have a detrimental effect on microorganisms. Ozone can oxidize lipids in the cell wall of microorganisms and penetrate the membrane to destroy ring DNA. This effect has been observed in various bacteria, including *Escherichia coli*, *Mycobacterium tuberculosis*, and *Pseudomonas aeruginosa*, as well as hydrophilic and lipophilic viruses and hepatitis viruses [13,14]. However, some studies have reported a positive effect of low doses of ozone on microorganisms. According to a study conducted in 2018, a concentration of 0.3–0.5 mg/mL of ozone can stimulate the growth of microorganisms [13,14].

Microorganisms are responsible for producing approximately 70% of the all-natural products that can be widely used in medicine, pharmacology, and agriculture. Among all microorganisms capable of synthesizing natural products, actinobacteria are particularly important [15,16,17,18,19]. Actinobacteria living in extreme conditions have been of particular interest. Extremophiles are organisms that can exist and reproduce in extreme environmental conditions, for example, high temperature and highly acidic environments [20]. In addition, high oxygen content may be an important factor for the growth and development of some rare groups of bacteria.

Microorganisms from ancient and unexplored ecosystems are of particular interest for scientific research. Lake Baikal is one such ecosystem, which is an established UNESCO World Heritage Site characterized by cold, low-mineralized water with high amounts of dissolved oxygen [21,22]. However, despite the great prospects in this area, only a few applied microbiological studies have been conducted on Lake Baikal [23,24,25,26,27]. In addition, the adaptation potential of Baikal microorganisms to pure oxygen has never been published and described.

We hypothesized that a unique group of extremophile microorganisms exists in Lake Baikal. They live with high amounts of oxygen and can feel hyperoxic effects in the wintertime. They probably have specific protective mechanisms against dissolved oxygen and ROS by producing both known and new natural products with antioxidant activity. It is assumed that littoral Baikal endemic invertebrates and their symbiotic/associated microbiota in wintertime can be adapted to the high oxygen content in water induced by algae blooms. Most continental reservoirs are characterized by an oxygen content ranging from 7 to 9 mg/L. Lake Baikal is characterized by a year-round oxygen content in its water ranging from 12 to 14 mg/L [21,28]. The high level of dissolved oxygen is spread on the water column, being approximately 12.75–12.94 mg/L at the depth profile of 0–25 m. Even at the depth of 1.350 m, the oxygen content is 9.56–9.63 mg/L [29]. During the under-ice algae bloom, local increases in oxygen concentration of up to 18 mg/L were observed and described by Prof. G.I. Galaziy [30]. In addition, during our environmental monitoring, we observed the concentration to be up to 21 mg/L in the shallow water littoral zone (unpublished data, Figure S1).

The aim of this study was to evaluate the impact of increased oxygen and ozone levels on the morphological characteristics and antioxidant capacity of actinobacteria derived from Lake Baikal.

## 2. Materials and Methods

### 2.1. Experimental Design

To evaluate the impact of oxygen and ozone on the morphological parameters of actinobacteria, antioxidant synthesis, and DNA mutations, the following experiments were conducted. Pure cultures of actinobacteria were isolated on solid nutrient media under conditions of high oxygen. The microorganisms were identified by the sequencing of the 16S rRNA gene. Then, the actinobacteria strains were cultured under three different conditions: a condition of natural aeration (control condition), an increased oxygen content in the Erlenmeyer flask (Experimental Condition 1), and an increased oxygen and ozone content in the Erlenmeyer flask (Experimental Condition 2). After every 7 days, samples were fixed for further assessment of morphological changes, extraction of secondary metabolites to assess antioxidant and antibiotic activities, analyze biotechnological potential with liquid chromatography and high-resolution mass spectrometry, and search for DNA mutations. The experimental scheme is shown in the Supplementary Materials (Figure S2).

### 2.2. Sampling, Location, and Isolation of Actinobacteria

Phytophagous amphipods *Eulimnogammarus cyaneus* were collected at a depth of 0–0.5 m from the southern part of Lake Baikal, near the Bolshoe Goloustnoe settlement. The sampling was performed in wintertime, under ice. The collection of amphipods was performed using traps. The concentration of dissolved oxygen in the ice hole was 15.4 mg/L. A moderate growth of macrophytes was observed in the sampling point. The collected amphipods were rinsed with 70% ethanol and sterile water, and then homogenized in a 20% sterile glycerol solution.

Two strains of actinobacteria belonging to the genera *Streptomyces* sp. and *Rhodococcus* sp. were isolated from the Baikal endemic amphipods. The isolation of actinobacterial strains was performed on solid nutrient MS media (containing soy flour—20 g/L, D-mannitol—20 g/L, agar—20 g/L) and TSB media (containing casein peptone (pancreatic)—17 g/L, dipotassium hydrogen phosphate—2.5 g/L, glucose—2.5 g/L, NaCl—5 g/L, soy peptone—3 g/L). The isolation and cultivation of microorganisms were carried out in a thermostat at 28 °C, using an oxygen generator Armed 7F-5L (Russia, Moscow) to maintain an oxygen level of 70% inside the thermostat chamber. The control conditions involved the isolation and cultivation of microorganisms without the use of oxygen generators. Strains similar to actinobacteria were identified based on colony morphology. The colonies were transferred from the primary plates to fresh MS or TSB plates to obtain a pure culture of each colony.

### 2.3. 16S rRNA Gene Sequencing and Analysis

Total DNA was isolated using the method described in [31]. Amplification of the 16S ribosomal RNA gene was conducted with primers 27F (AGA GTT TGATYM TGG CTC AG) and 1510R (TAC GGY TAC CTT GTT ACG ACT T). PCR products were detected in 1% agarose gel using ethidium bromide, purified with a Cleanup Standard PCR purification kit (Kat. BC022, Evrogen, Russia, Moscow). PCR was carried out in a TGradient Thermocycler (Biometra, Germany, Göttingen) at a volume of 25 μL. The PCR parameters were as follows: initial denaturation at 95 °C for 5 min, followed by 25 cycles of 95 °C for 40 s, 49–52 °C for 25 s, and 72 °C for 110 s, and final elongation at 72 °C for 5 min [32]. Forward and reverse sequences were assembled using the Bioedit software (version 7.2.5). The sequences were deposited in the GenBank (OR047882, OR047883). Evolutionary history was inferred using the neighbor-joining method [33]. The nucleotide sequences from the experiment were aligned with sequences with the greatest similarity from the NCBI database. Evolutionary distances were computed using the Tamura–Nei method [34]. The evolutionary analysis was conducted using MEGA X [35].

### 2.4. Cultivation of Actinobacteria

The strains were cultivated in liquid nutrient NL19 media (soy flour—20 g/L, D-mannitol—20 g/L). Experimental Condition 1 involved cultivation under increased oxygen content up to 24.3 mg/L in the Erlenmeyer flask. Experimental Condition 2 involved cultivation under increased oxygen (24.3 mg/L) and ozone content. For Experimental Condition 2, the nutrient medium was ozonated for 15 min each day. The oxygen level was determined using the MARK-303-T industrial portable dissolved oxygen analyzer (Russia, Nizhniy Novgorod). Ozone was generated using the Vosoco AZ-1G-G ozone generator (China) at a rate of 1000 mg/h. The control group consisted of actinobacteria cultivated under conditions of natural aeration, where the oxygen content was 7.3 mg/L. Inoculation of pure cultures was carried out in sterile Erlenmeyer flasks with glass balls. Each 500 mL flask was filled with 250 mL of nutrient media. Cultivation was conducted in orbital shaker DOS-20L (Elmi, Latvia, Riga) at 28 °C with a shaking rate of 120 rpm for 3 months. Every 7 days, the actinobacteria were transferred to flasks with fresh media.

### 2.5. Microscopy of the Strains

The cells of microorganisms were colored using the Gram method [36]. Microscopy was performed using an immersion system at a magnification of 1000× on a bright-field microscope Mikromed-3–20 (Russia, Saint-Petersburg), equipped with a digital camera.

### 2.6. Searching for DNA Mutations

Multiple alignments of nucleotide sequences were performed to detect mutations. For this aim, the standard parameters of the ClustalW algorithm in the MEGA X software package were used [35]. Only samples with high-quality chromatograms of nucleotide sequences were included in the analysis. To detect the localization of DNA mutations, our sequences were aligned to reference sequence NR_024570.1 (NCBI, 16S rRNA *E. coli*).

### 2.7. Extraction of Secondary Metabolites

The cultures grown in flasks were transferred into sterile 50 mL Falkon tubes. The grown cultures were centrifuged at 3000 rpm for 10 min on centrifuge Armed LC-04B (Armed, Russia, Moscow) to separate the biomass and cultural liquid. The culture fluid metabolites were extracted with an equal volume of isobutanol (Vecton, Russia, Saint-Petersburg). The metabolites from the bacterial biomass were extracted with 10 mL of isobutanol. The resulting mixture was sonicated for 15 min and shaken for 1 h, followed by centrifugation at 3000 rpm for 10 min [37]. The obtained crude extracts were weighed and dissolved in a mixture of methanol:DMSO (1:1) at a concentration of 10 mg/mL for further evaluation of antioxidant activity and antibiotic effects against test cultures, as well as subsequent HPLC–MS analysis.

### 2.8. Antioxidant Activity Assay of Extracts from Isolated Strains

The qualitative antiradical activity of actinobacteria extracts was determined using the 2,2-diphenyl-1-picrylhydrazyl (DPPH) test [31,38]. DPPH (0.0236 g/L) was dissolved in methanol. DPPH and samples were mixed in a 96-well plate. DPPH and samples were mixed in a proportion of 9:1. The plate was incubated for 30 min in darkness. After 30 min, a color change was assessed. A change from pink to yellow indicated the presence of antioxidants in the samples. Spectrophotometric analysis was performed using a microplate reader Infinite M200 (Tecan, Austria, Männedorf), at a wavelength of 517 nm. Dissolved DPPH and methanol were used as a negative control.

### 2.9. Antibiotic Assay of Extracts from Isolated Strains

The antibiotic activity was tested using the disk diffusion method [39]. Two strains of microorganisms, *Bacillus subtilis* ATCC 66337 and *Pseudomonas putida* KT 2440, were chosen as model test cultures. Overnight test cultures were inoculated on solid nutrient LB media (tryptone—10 g/L, yeast extract—5 g/L, NaCl—5 g/L) and dried at room temperature. A total of 45 μL of cultural liquid or biomass extracts was loaded onto 6 mm paper disks and dried at room temperature. The incubation of plates with disks was held throughout 24 h at 37 °C. The zones of inhibition were measured with ±1 mm accuracy [40,41].

### 2.10. Liquid Chromatography–Mass Spectrometry (LC–MS) and Dereplication Analysis

The biotechnological potential of the biomass and cultural liquid extracts was evaluated using HPLC approaches. The Thermo Fisher Scientific Ultimate 3000 chromatography system (Dionex, Waltham, MA, USA) was coupled with a high-resolution Maxis II mass spectrometric detector Q-TOF maXis Impact II (Bruker Daltonics, Fremont, CA, USA). A linear gradient of solvents (water: acetonitrile) from 5% to 95% was used for separation over 18 min with a C18 column (Acquity UPLC BEH, Framingham, MA, USA) of 130 Å, 1.7 µm, and 2.1 mm × 100 mm [15]. Mass detection was performed in a positive mode, with the detection range set to 160–2500 *m*/*z*. In order to minimize the fragmentation of natural products during analysis, we used a gentle ionization. ESI ionization with low ionization energy was used. This part of the study was conducted at Helmholtz-Institut für Pharmazeutische Forschung Saarland (HIPS) and Saar university. Dereplication was carried out using the Dictionary of Natural Products database (CRC-press. v.10.1 2019) with the search parameters of accurate molecular mass (MM) and biological source—*Streptomyces* or *Rhodococcus*. Natural products were identified according to the basic principles of dereplication—when the difference between the accurate mass was less than m/z 0.001 and 10 ppm and the biological source was identical with library data, simultaneously. The masses of the all-natural products were calculated using the standard adducts ([M + H] ^+^, [M + Na] ^+^, [M+ NH4] ^+^, etc.) [42]. The analysis of each sample was carried out three times.

## 3. Results

### 3.1. Isolation of Actinobacteria in Standard and Experimental Conditions

It was observed that the first actinobacteria-like strains appeared on the fourth day of the experiment under the control group (natural aeration conditions). However, in Experimental Condition 1 (an increased oxygen content), the first colonies of actinobacteria-like strains appeared on the third day of the experiment. The mentioned strains were identified based on colony morphology. As a result, during the experiment, 47 actinobacteria cultures were isolated under high oxygen content. In conditions of natural aeration, only three actinobacteria cultures were isolated. Thus, we demonstrated that oxygen influences the isolation and diversity of actinobacteria strains.

### 3.2. 16S rRNA Gene Sequencing and Analysis

Among all isolated microorganisms, two strains of actinobacteria *Streptomyces* sp. LPB2020M1 and *Rhodococcus* sp. LPB2020M2 were identified by 16S rRNA gene sequencing. These strains were chosen as representatives of contrasting genera.

**Figure 1:**
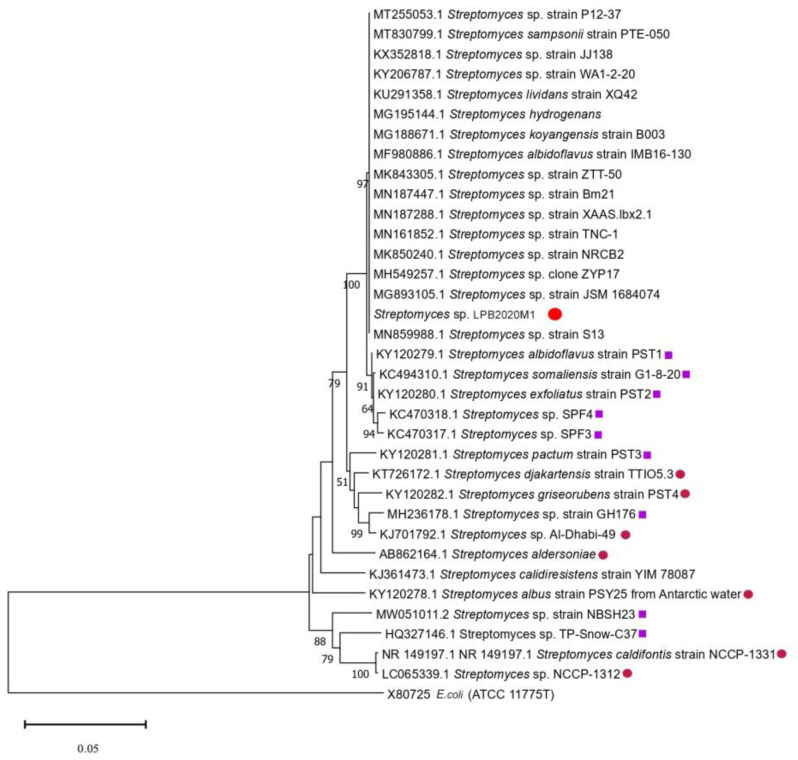
Evolutionary relationships of the Baikal strain *Streptomyces* sp. The strain isolated during the experiment is marked with a bold red circle «
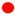
». The strains isolated from cold sources are marked with a purple square «
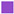
». The strains isolated from hot sources are marked with a pink circle «
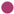
».

Figure 1 shows the phylogenetic tree of the strain *Streptomyces* sp. LPB2020M1. Based on the figure, it can be seen that the strain is in a clade with other representatives of the genus *Streptomyces*, such as *Streptomyces sampsonii*, *Streptomyces albidoflavus*, and *Streptomyces hydrogenans*.

**Figure 2:**
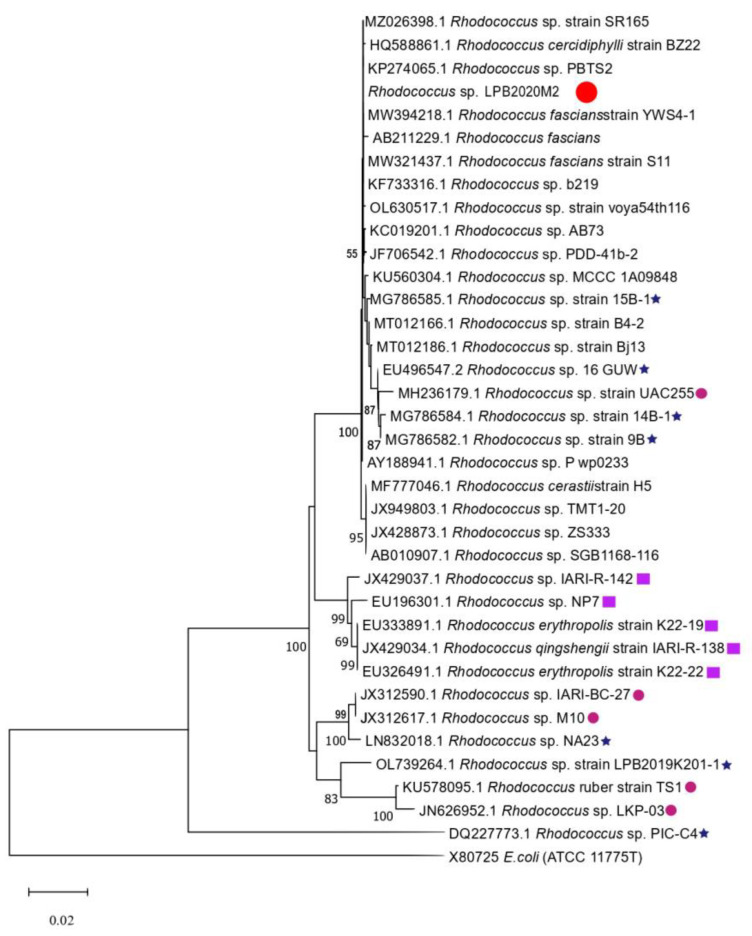
Evolutionary relationships of the Baikal strain *Rhodococcus* sp. The strain isolated during the experiment is marked with a bold red circle «
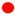
». The strains isolated from cold sources are marked with a purple square «
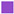
». The strains isolated from hot sources are marked with a pink circle «
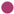
». The strains isolated from Lake Baikal are marked with a blue star «
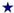
».

Figure 2 shows the phylogenetic tree of the strain *Rhodococcus* sp. LPB2020M2. In this case, the clades are quite mixed. The isolated strain does not form a separate clade with other representatives of the genus *Rhodococcus*.

### 3.3. Assessment of Morphological Changes

**Figure 3:**
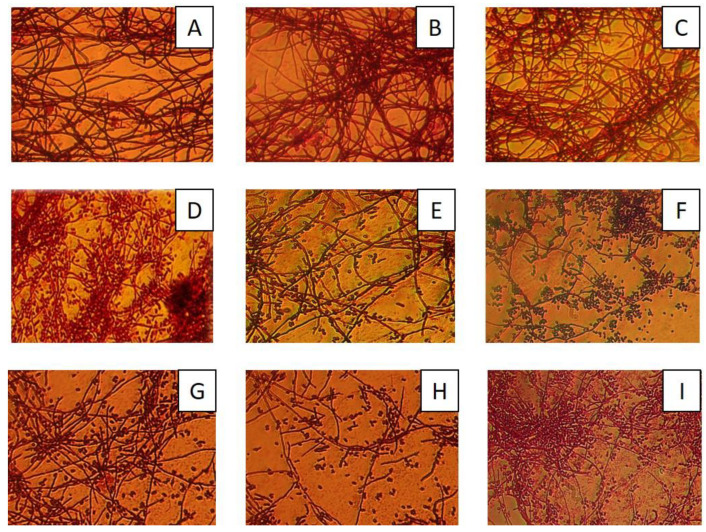
Morphological changes of *Streptomyces* sp. LPB2020M1 grown in control and experimental conditions. (**A**) Exposure to conditions of natural aeration, beginning of the experiment (Week 1); (**B**) exposure to conditions of natural aeration, middle of the experiment (Week 5); (**C**) exposure to conditions of natural aeration, end of the experiment (Week 10); (**D**) exposure to conditions of increased oxygen content, beginning of the experiment (Week 1); (**E**) exposure to conditions of increased oxygen content, middle of the experiment (Week 5 s); (**F**) exposure to conditions of increased oxygen content, end of the experiment (Week 10); (**G**) exposure to conditions of increased oxygen and ozone content, beginning of the experiment (Week 1); (**H**) exposure to conditions of increased oxygen and ozone content, middle of the experiment (Week 5); (**I**) exposure to conditions of increased oxygen and ozone content, end of the experiment (Week 10).

During the experiment on the cultivation of actinobacteria in liquid nutrient media under standard and experimental conditions, an increase in the sporulation rate of strain *Streptomyces* sp. LPB2020M1 was observed in both experimental groups. In addition, the cultivation of *Streptomyces* sp. LPB2020M1 in conditions of natural aeration did not lead to effects of sporulation during the whole time of the experiment (Figure 3). No visible effects were observed for the strain *Rhodococcus* sp. LPB2020M2 growing in experimental conditions.

### 3.4. DNA Mutations in 16S rRNA Gene

We were assessing the mutagenic effects of oxygen and ozone on the genetic apparatus of the strains *Rhodococcus* sp. LPB2020M2 and *Streptomyces* sp. LPB2020M1. The mutagenic effect was evaluated for the gene 16S rRNA.

**Figure 4:**
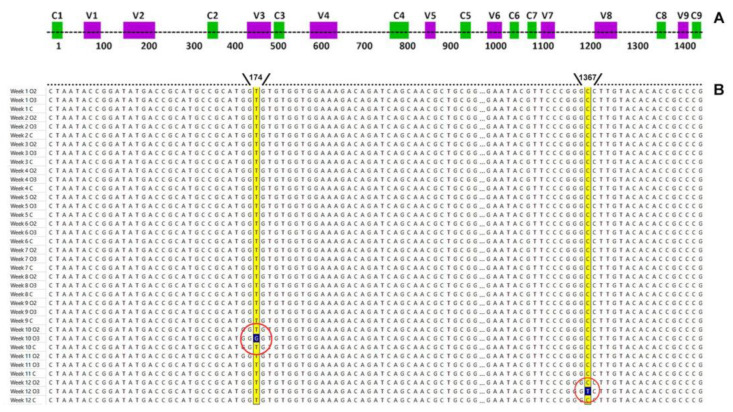
Representative picture of the variable (purple) and conserved (green) regions in the 16S rRNA gene and their locations (**A**) [43], and multiple alignment and nucleotide substitutions in the 16S rRNA gene (**B**). The mutation sites are marked with a red circle for *Rhodococcus* sp. LPB2020M2.

For *Rhodococcus* sp. LPB2020M2, nucleotide substitutions were revealed in two experimental samples (exposure to oxygen and ozone) at 10 and 12 weeks. In the 10th week of the experiment, thymine was replaced with guanine, while in the 12th week, cytosine was replaced with thymine (Figure 4). All mutations have been found in the variable V2 and V9 regions of the conservative gene.

Nonetheless, no nucleotide substitutions were detected in the control group during the whole experiment. At the same time, no damage or spontaneous DNA mutation was detected for the *Streptomyces* sp. LPB2020M1 strain.

### 3.5. Antioxidant Activity of Extracts from Isolated Strains

During the analysis of cultural liquid extracts of *Streptomyces* sp. LPB2020M1, antioxidant activity was observed in all experimental samples throughout the duration of the experiment. In the control group, an increase in antioxidant activity was noted only once, specifically in the third week.

The analysis of biomass extracts showed an increase in antioxidant activity in all experimental group samples during most weeks, with the exception of one extract (ozone) in the fifth week. Similar to the cultural liquid extracts, the control group samples exhibited an increase in antioxidant activity during the third week of the experiment.

However, the analysis of biomass and cultural liquid extracts of the strain *Rhodococcus* sp. LPB2020M2 in the experimental group did not show an increase in antioxidant activity. Table 1 provides the antioxidant activity data for *Streptomyces* sp. LPB2020M1.

### 3.6. Antibiotic Activity of Extracts from Isolated Strains

Crude metabolite extracts from isolated actinobacterial strains were tested for antibiotic activity. We demonstrated the presence of antimicrobial activity in the cultural liquid experimental extracts of *Streptomyces* sp. LPB2020M1 from the 5th to the 11th weeks against *Bacillus subtilis*. In contrast, in the control conditions, antimicrobial activity was observed only once, after 7 days of cultivation.

Regarding the biomass extracts, antimicrobial activity was observed only once, specifically after 11 weeks of the experiment, in the sample exposed to oxygen and ozone aeration. Table 2 illustrates the antimicrobial activity of *Streptomyces* sp. LPB2020M1 against *Bacillus subtilis*. No antibiotic activity against *Pseudomonas putida* was observed throughout the entire experiment.

Nonetheless, no antibiotic activity was noted for *Rhodococcus* sp. LPB2020M2.

### 3.7. Dereplication of the Secondary Metabolites

**Figure 5:**
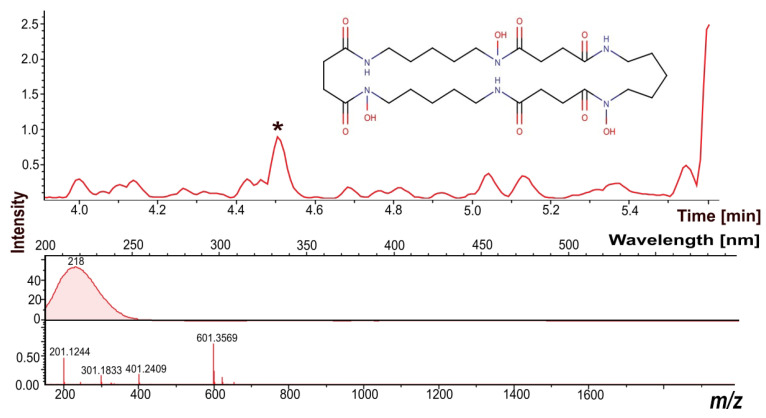
Mass chromatogram and UV and mass profiles of the natural product identified as the antioxidant Nocardamine. “*” indicates a peak of natural product.

The experiment aimed to assess the effects of dissolved oxygen and ozone on the oxidative adaptive abilities of actinobacterial strains. Several identified natural products were found, which may be responsible for antioxidative activity. Among these natural products, one was characterized by a molecular mass (MM) of 600.3486 Da (retention time (RT) 4.5 min, 0.534 ppm), and it was identified as the antioxidant Nocardamine (Figure 5). Nocardamine is known to possess antioxidant, siderophore, and antibiotic properties [44].

**Figure 6:**
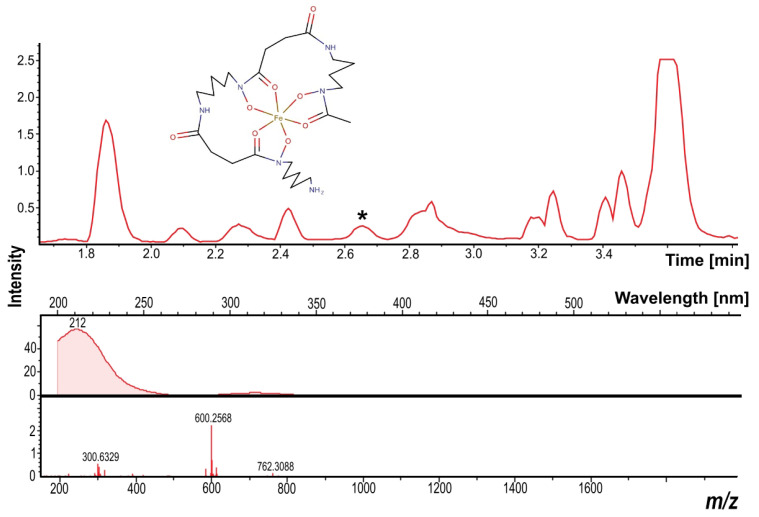
Mass chromatogram and UV and mass profiles of the natural product identified as Ferrioxamine A2. “*” indicates a peak of natural product.

Figure 6 shows the synthesis of Ferrioxamine A2 (RT—2.6 min, MM—599.2496 Da, ppm—0.537) in both experimental conditions. This natural product is an iron carrier [45].

Furthermore, a molecule characterized by the MM of 707.5335 Da (RT 6.9 min) was synthesized under experimental conditions and was found once during the third week of the control group. Considering that antioxidant activity was specifically detected during the third week in the control conditions, we can assume that this natural product possesses antioxidant activity.

Upon analyzing the chromatograms of *Streptomyces* sp. LPB2020M1, two unknown natural products were detected. The first molecule, with a molecular mass of 572.3175 Da (RT 4.0 min), was synthesized only under experimental conditions and was not detected in the control samples. The second molecule, with a mass of 751.3685 Da (RT 15.3 min), was detected in all experimental samples except for one during the fifth week of the experiment (ozone exposure). These compounds were synthesized by the strain only under experimental conditions and, therefore, may be antioxidants. According to the results of the DPPH test, antioxidant activity was detected in all experimental samples, except for the sample exposed to ozone during the fifth week of the experiment.

**Figure 7:**
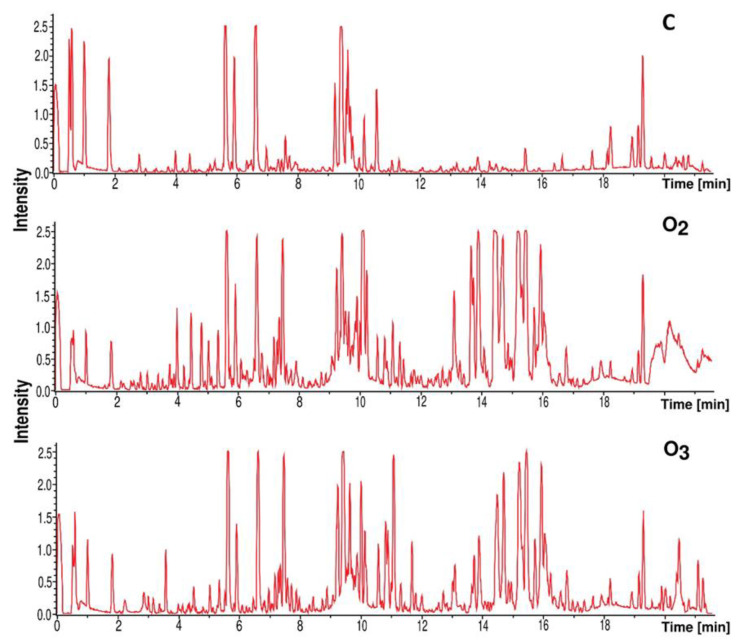
Effect of experimental conditions on natural products synthesized by *Streptomyces* sp. LPB2020M1. O_2_—exposure to increased oxygen content; O_3_—exposure to increased oxygen and ozone content; C—control (natural aeration).

There was an induction of the synthesis of the natural products compared to the natural aeration conditions from the RT from 10.6 to 17 (Figure 7). At this time interval, a molecule named Streptomyceamide B (MM—264.0747 Da, ppm—1.2) was identified. Other natural products, such as Arginomycin (MM—436.2219 Da, RT—10.8, ppm—8.3), Nivelactam (MM—439.2704 Da, RT—12.1, ppm—4.2), Antimycin A (MM—440.3655 Da, RT—13.7, ppm—1.7), 5,10,11-Trihydroxy-3-cadinanone (MM—270.1833, RT—12.7, ppm—0.703), 11-Methyl-2-tridecanone (MM—212.2139 Da, RT—14.2 min, ppm—0.541), and 10-Methylhexadecanoic acid (ξ)-form (MM—270.2561 Da, RT—14.3 min, ppm—0.814) were also detected at this time interval.

**Figure 8:**
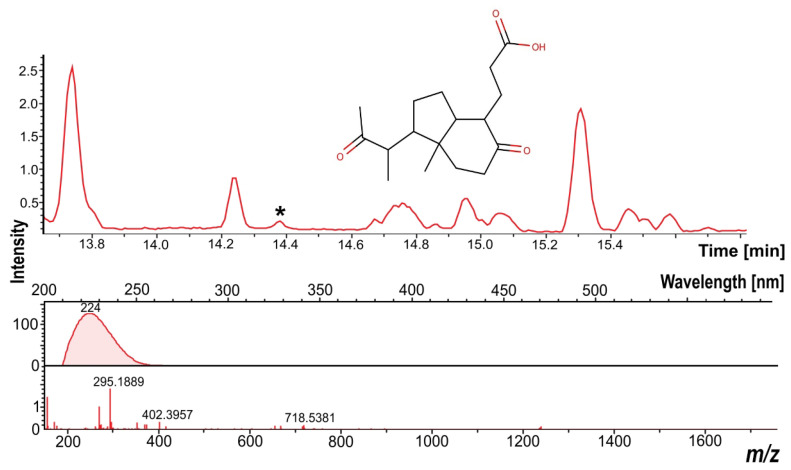
Mass chromatogram and UV and mass profiles of the natural product identified as Octahydro-7a-methyl-1-(1-methyl-2-oxopropyl)-5-oxo-1H-indene-4-propanoic acid. “*” indicates a peak of natural product.

For *Rhodococcus* sp. LPB2020M2, natural products with antioxidant activity were not detected in any of the experimental weeks. However, it was determined that under both the control and experimental conditions, *Rhodococcus* sp. LPB2020M2 synthesizes at least 89 natural products. Out of these, 88 molecules were not identified using the Dictionary of Natural Products database and the used parameters of dereplication. The only one known natural product was identified as Octahydro-7a-methyl-1-(1-methyl-2-oxopropyl)-5-oxo-1H-indene-4-propanoic acid (MM—294.181 Da, RT—14.4, ppm—7.172) (Figure 8). Based on the information available in the DNP database, this molecule exhibits antifungal activity.

## 4. Discussion

We performed an experiment aimed at demonstrating the impact of oxygen and ozone on the morphological parameters of actinobacteria, their antioxidant synthesis, and their DNA mutations. The classification of bacteria and differences between Gram-positive and Gram-negative forms are well-known. In addition, the attitude of bacteria to air is well-known, manifesting to grow and develop in aerobic or anaerobic conditions. However, there is little research on the role of the effects of different gases included in the content of air. Such research may be relevant for microorganisms whose habitat is in air conditions, for example, in the atmosphere. The content of oxygen in air is relatively stable and is approximately 20.9%. Other gases are nitrogen (approximately 78%), argon (approximately 0.9%), and carbon dioxide (approximately 0.03%), with other gases in smaller concentrations [46].

The content of dissolved oxygen In freshwater reservoirs varies from 0 to 12 mg/L. Typically, the concentration of dissolved oxygen in concentrations higher than 14 mg/L can be achieved in the saturation of water in conditions of low temperatures, low organic content, and the covering of a water reservoir by ice in the winter period. There are only several natural locations on the planet where oxygen oversaturation can exist, such as the freshwater lakes of Antarctica (such as Lake Vostok and Radok Lake) and Lake Baikal. The microbial compositions of Lake Vostok and Radok Lake have been published and partially compared with the microbial composition of Lake Baikal, where a common group of bacteria related to the *Actinobacteria* phylum was detected [47,48,49,50]. In the mentioned lakes, we probably have to deal with a unique group of extremophilic bacteria related to oxyphiles.

The concept of “oxyphilic bacteria” has undergone significant transformation during the 20th and 21st centuries and is generally used in relation to aerophilic and aerobic microorganisms without specific reference to oxygen as an environmental factor. Nowadays, mentions of terms such as “oxyphilic bacteria” or “oxyphobic bacteria” are extremely rare in the literature, despite the fact that adaptations to oxygen as an environmental factor are crucial for under-ice communities. The attachment to “oxyphilia” has gained widespread use in cellular biology when characterizing different cell types. However, in microbiology, this concept is nontrivial and rarely mentioned [51,52].

Despite the ecological and evolutionary significance of oxygen in biological systems, there are currently limited references in scientific and technical sources regarding the effects of dissolved oxygen and ROS on microorganisms. For instance, it has been described that an increase in the concentration of dissolved oxygen leads to an elevated oxygen gradient, which in turn enhances the delivery of oxygen to the cells of microorganisms [53]. 

Recent studies have shown that oxidative stress is a major cause of many human diseases [54]. For example, the association between decreased reproductive potential in humans and oxidative stress has been repeatedly demonstrated [55]. ROS affect the dynamic processes of gamete maturation, transportation through the reproductive tract, and fertilization, as well as the subsequent development and implantation of preimplantation embryos [56,57]. Currently, there is sufficient evidence about the effect of oxidative stress and ROS on orphan diseases and on the nervous system, such as amyotrophic lateral sclerosis and chronic demyelinating polyneuropathy [58,59,60]. Thus, the search for biologically active molecules is especially relevant, as they can serve as potential prototypes of new drugs for diseases associated with oxidative stress. The members of the genus *Streptomyces* are bacteria well-known for their ability to produce a wide range of secondary metabolites used in human and veterinary medicine [61,62,63]. 

In the current study, we demonstrated that actinobacteria cultivated under experimental conditions exhibited an elevated level of sporulation compared to natural aeration conditions throughout the 3-month experiment. This observation confirms the development of protective adaptations to high oxygen levels and oxidative stress in the environment. Considering that these adaptations are accompanied by changes in metabolism and the synthesis of secondary metabolites, increasing the oxygen content during actinobacteria cultivation can be utilized to induce the production of secondary metabolites.

Therefore, due to the close relationship between the oxidative stress of the aggressive environment and the activation of defense mechanisms and synthesis of natural compounds, the project envisaged the highly probable isolation of known and novel antioxidants.

The analysis of secondary metabolites demonstrated that the exposure of the strain *Streptomyces* sp. LPB2020M1 under experimental conditions leads to the synthesis of at least three new (not mentioned in the DNP database) natural products. In addition, we demonstrated an oxygen-related induction of the synthesis of Arginomycin, Nivelactam, Antimycin A, Streptomyceamide B, 5,10,11-Trihydroxy-3-cadinanone, Hygroscopin B, and 11-Methyl-2-tridecanone.

New antioxidants are often synthesized by actinobacteria isolated from aquatic invertebrates. Such examples are the family of actinoporins synthesized by *Actinokineospora* sp. isolated from the sponge *Spheciospongia vagabunda* [64]. Another example is the antioxidant Ageloline A, which is synthesized by a widespread strain of *Streptomyces* sp. isolated from the sponge *Agelas oroides* [65]. *Nocardiopsis* sp. PU3, isolated from the coral reef of Pullivasal Island, was found to be a potential source of antioxidants [66].

In our study, the natural product, identified as Nocardamine, also known as Desferrioxamine E, belongs to the class of hydroxamates and has been identified as an antioxidant. Additionally, under both experimental conditions, the synthesis of Ferrioxamine A2, a molecule known to function as an iron carrier, was observed. Iron is known to contribute to the formation of ROS, and various iron chelators are used in medicine to mitigate its effects [67,68]. In a study by Lan, it was demonstrated that the administration of desferrioxamine significantly reduces iron levels in the brain in mouse models of Parkinson’s disease [45,69]. Here, the synthesis of the mentioned molecules was observed as a response of bacteria to oxidative stress.

The influence of the experimental conditions on the genetic apparatus was observed. DNA mutation analysis of the strain *Rhodococcus* sp. LPB2020M2 revealed two nucleotide substitutions during the later stages of experimental exposure in a nutrient medium with oxygen and ozone. ROS often contribute to nucleotide substitutions, such as from thymine to cytosine, guanine to cytosine, and thymine to adenine [70]. Based on this observation, it can be inferred that high concentrations of oxygen in the environment can have a mutagenic effect on the genetic apparatus of microorganisms. On the other hand, no damage or spontaneous DNA mutation was detected in *Streptomyces* sp. LPB2020M1. This may be attributed to the synthesis of Nocardamine (Desferrioxamine E) by this strain. Previous studies have shown that desferrioxamine protects DNA against oxidative damage by chelating redox-active iron present in the endosomal–lysosomal compartment [71].

In addition, in the current study, we observed that nucleotide substitutions occurred in variable regions of a highly conserved gene. It is worth noting that the mutagenic effect can also be influenced by the GC content, as GC base pairs are characterized by the presence of three hydrogen bonds. Actinobacteria, including *Streptomyces* and *Rhodococcus*, are known to have a high GC content. The GC content in *Streptomyces* species is approximately 74%, which is slightly higher than that in *Rhodococcus* species, with a range from 61% to 71% [72,73].

These findings demonstrate the impact of dissolved oxygen on Lake Baikal microorganisms, which are naturally adapted to its high oxygen content. Additionally, we observed species-specific reactions among the strains. Therefore, we demonstrated that microorganisms in Lake Baikal have the capability to synthesize novel natural products as new metabolites and markers of stress response. Considering the regressive dynamics of discovering new classes of natural products [74], it is presumed that addressing the global challenges facing global healthcare and biomedicine necessitates the search for microorganisms that serve as producers of novel biologically and pharmaceutically active natural products. Lake Baikal provides a unique opportunity to simultaneously study psychrophilic and oxyphilic microorganisms and assess their adaptive and biosynthetic potential for pharmacy use.

## Figures and Tables

**Table 1:** Results of the DPPH test.

	Cultural Liquid						Biomass					
*Streptomyces* sp. LPB2020M1	Week No						Week No					
	1	3	5	7	9	11	1	3	5	7	9	11
O_2_	+	+	+	+	+	+	+	+	+	+	+	+
O_3_	+	+	−	+	+	+	+	+	−	+	+	+
C	−	+	−	−	−	−	−	+	−	−	−	−

O_2_—exposure to increased oxygen content; O_3_—exposure to increased oxygen and ozone content; C—control (natural aeration). “+” indicates the presence of antioxidant activity, “−” indicates the lack of antioxidant activity.

**Table 2:** Antimicrobial activity of *Streptomyces* sp. LPB2020M1 when grown in different conditions.

	Cultural Liquid						Biomass					
*Streptomyces* sp. LPB2020M1	Week No						Week No					
	1	3	5	7	9	11	1	3	5	7	9	11
O_2_	−	−	+	+	+	+	−	−	−	−	−	−
O_3_	−	−	+	+	+	+	−	−	−	−	−	+
C	+	−	−	−	−	−	−	−	−	−	−	−

O_2_—exposure to increased oxygen content; O_3_—exposure to increased oxygen and ozone content; C—control (natural aeration). “+” indicates the presence of antibiotic activity, “−” indicates the lack of antibiotic activity.

## Data Availability

All data presented in this research are available in the main body of the manuscript.

## Supplementary Materials

The following supporting information can be downloaded at: https://www.mdpi.com/article/10.3390/metabo13070830/s1, Figure S1. Photography of oxymeter, demonstrating a high environmental rate of dissolved oxygen. Figure S2. Experimental design scheme.
