# Supplementary figures and images for: The Effects of a High Concentration of Dissolved Oxygen on Actinobacteria from Lake Baikal
Source: Metabolites. 2023 Jul 7;13(7):830. doi: 10.3390/metabo13070830 (PMC10386110; doi:10.3390/metabo13070830)

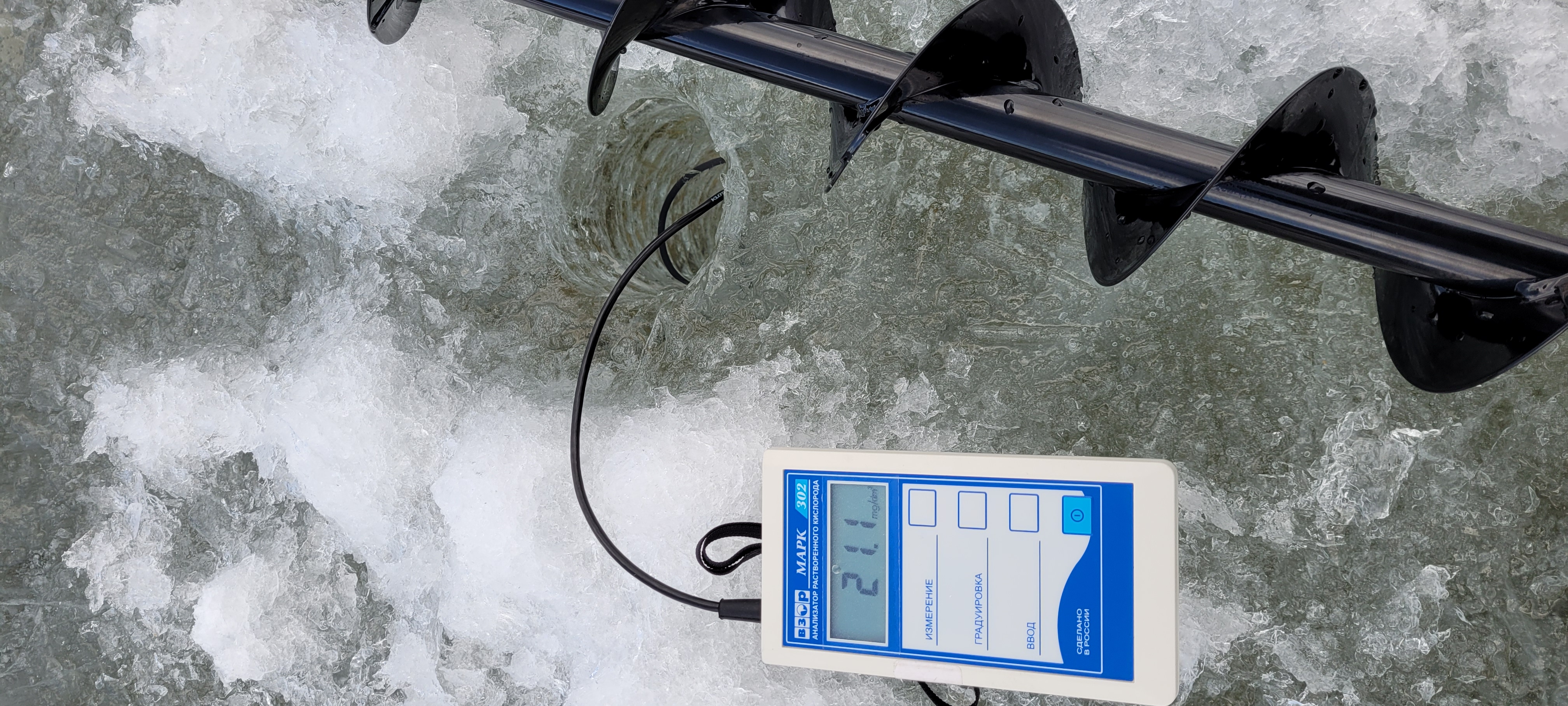

Supplement: Supplementary file 1 [file metabolites-13-00830-s001.zip › S1.jpg]

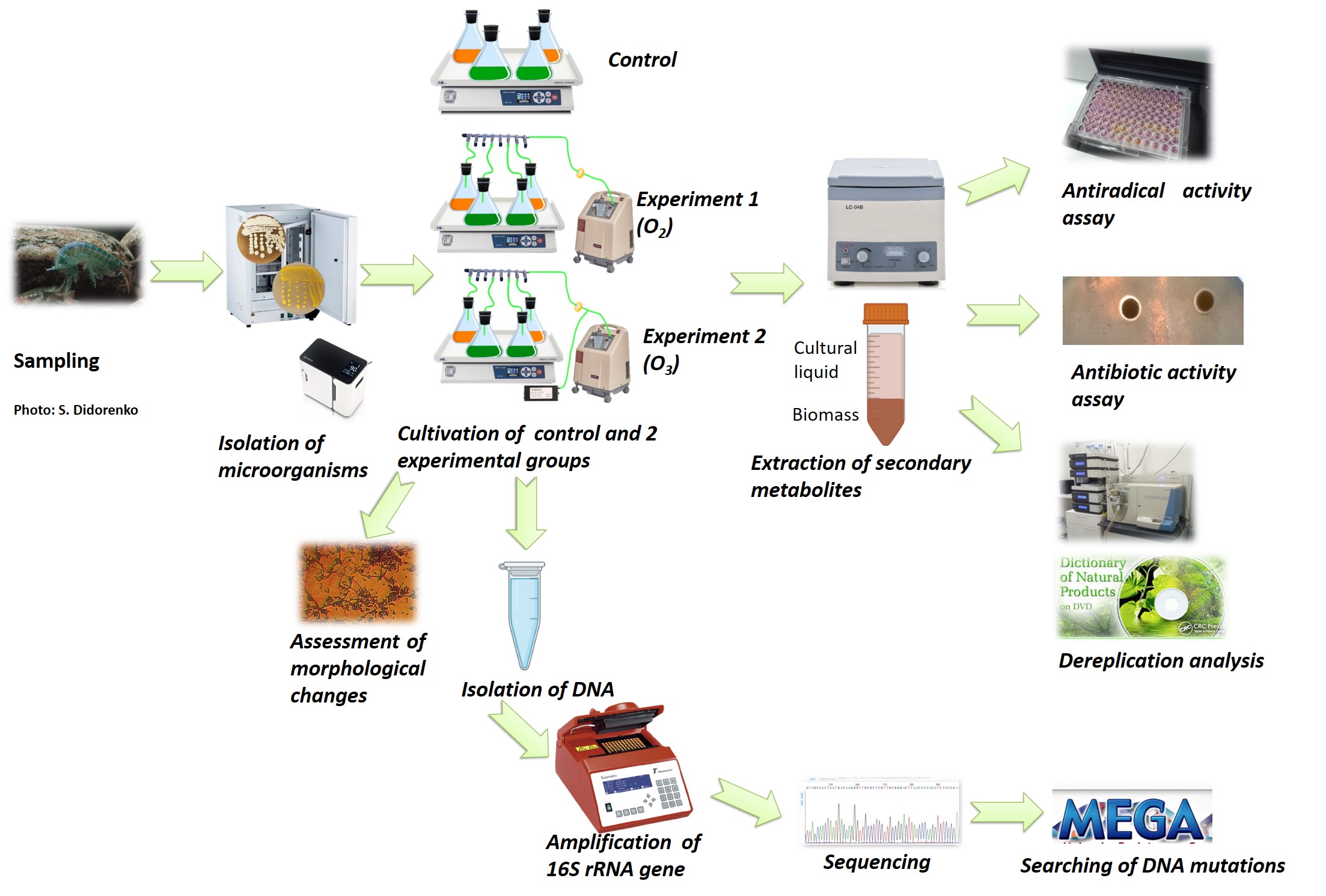

Supplement: Supplementary file 1 [file metabolites-13-00830-s001.zip › S2.jpg]
